# Mitochondrial function parameters as a tool for tailored drug treatment of an individual with psychosis: a proof of concept study

**DOI:** 10.1038/s41598-020-69207-4

**Published:** 2020-07-23

**Authors:** Tamara Bar-Yosef, Wessal Hussein, Ofer Yitzhaki, Odeya Damri, Limor Givon, Carmit Marom, Vlada Gurman, Joseph Levine, Yuly Bersudsky, Galila Agam, Dorit Ben-Shachar

**Affiliations:** 1 Department of Clinical Biochemistry and Pharmacology, Faculty of Health Sciences, Ben-Gurion University of the Negev and Mental Health Center, Beer Sheva, Israel; 2 Laboratory of Psychobiology, Department of Psychiatry, Rambam Health Care Campus, B. Rappaport Faculty of Medicine and Rappaport Family Institute for Research in Medical Sciences, Technion IIT, 31096 Haifa, Israel; 3 Mental Health Center, Beer Sheva, Israel; 4 Division of Psychiatry, Faculty of Health Sciences, Ben-Gurion University of the Negev and Mental Health Center, Beer Sheva, Israel

**Keywords:** Molecular neuroscience, Predictive markers

## Abstract

Pharmacological treatment of mental disorders is currently decided based on "trial and error" strategy. Mitochondrial multifaceted dysfunction is assumed to be a major factor in the pathophysiology and treatment of schizophrenia (SZ) and bipolar disorder (BD). This study aimed to explore the feasibility of using a profile of mitochondrial function parameters as a tool to predict the optimal drug for an individual patient (personalized medicine). Healthy controls (n = 40), SZ (n = 48) and BD (n = 27) patients were recruited. Mental and global state of the subjects, six mitochondrial respiration parameters and 14 mitochondrial function-related proteins were assessed in fresh lymphocytes following in-vitro or in-vivo treatment with five antipsychotic drugs and two mood-stabilizers. In healthy controls, hierarchal clustering shows a drug-specific effect profile on the different mitochondrial parameters following in-vitro exposure. Similar changes were observed in untreated SZ and BD patients with psychosis. Following a month of treatment of the latter patients, only responders showed a significant correlation between drug-induced in-vitro effect (prior to in-vivo treatment) and short-term in-vivo treatment effect for 45% of the parameters. Long- but not short-term psychotropic treatment normalized mitochondria-related parameters in patients with psychosis. Taken together, these data substantiate mitochondria as a target for psychotropic drugs and provide a proof of concept for selective mitochondrial function-related parameters as a predictive tool for an optimized psychotropic treatment in a given patient. This, however, needs to be repeated with an expanded sample size and additional mitochondria related parameters.

## Introduction

Schizophrenia (SZ) and bipolar-disorder (BD) share emotional and cognitive abnormalities and psychotic symptoms, commonly affecting young adults. Both disorders also share genetic risks and endophenotypes^1–3^. The research domain criteria (RDoC), based on observable behavioral (symptoms) and neurobiological dimensions rather than traditional diagnostic measures^4^ claims a lack of clear boundaries between SZ and BD. Henceforth, in the present study, we studied SZ and BD as a single entity^5^.

More than 40 different antipsychotic and mood stabilizing drugs are currently available. It has long been held that there is little difference in the therapeutic efficacy of antipsychotics other than clozapine and differences in clinical effects were mainly ascribed to variability in sedative and adverse effect profiles. The truth of this assertion has recently been challenged by meta-analyses, which found small but consistent differences between the drugs, corroborating a strong impression among clinicians that inter-individual differences in the efficacy of different antipsychotics do exist. Currently, there is no empirically-supported basis for the decision which psychotropic drug to prescribe to which patient in every day clinical practice. Choice is based on the clinical experience of the psychiatrist, on guidelines and on the individual patient’s clinical state, prior symptom response, and experience of side effects^6^.

Two core mitochondrial functions, energy production and Ca^2+^ homeostasis, are crucial for neuronal activity. Indeed, multifaceted mitochondrial dysfunction is evident by genetic, molecular, biochemical, structural and imaging studies in SZ and BD^7–9^. Changes in mitochondria-related intracellular pathways regulating neuronal transmission, plasticity, oxidative stress, apoptosis and cell survival have been observed in both disorders^10–16^. Hence, mitochondrial dysfunction can lead to distorted connectivity of neuronal networks and thereby to abnormal cognitive and emotional behaviors. In line with the latter, we have recently shown that transplantation of healthy mitochondria improved aberrant mitochondrial function, amended neuronal differentiation and synaptic connectivity in SZ-derived neurons and restored behavioral deficit in an animal model of the disorder^17^. Notably, mitochondria are also a target for psychotropic drugs by virtue of the drugs' modulatory effect on the expression of mitochondrial genes expression and on levels of proteins involved in the tricarboxylic acid cycle, the respiratory chain, apoptosis, autophagy and mitochondrial network dynamics^16,18–22^.

It has long been recognized that neuropsychiatric disorders associated with brain mitochondrial malfunction also present aberrant mitochondrial energetics in peripheral cells such as platelets, lymphocytes and leukocytes. In a broader scope, blood cells have been shown to retain a variety of biological abnormalities implicated in mental disorders and have been suggested as a proxy to brain tissue^23–27^.

Given the impairments observed in mitochondria in both disorders, together with the findings that mitochondria are a target for antipsychotics and mood stabilizers (jointly designated psychotropic drugs), we aimed to study the feasibility of using an array of mitochondrial parameters in blood lymphocytes as a tool to predict the optimal drug for an individual patient (personalized medicine). To this end the following issues had to be addressed: first, examine whether different drugs exert a specific response profile in-vitro in lymphocytes, which was studied in healthy subjects. Second, study the relevance of these in-vitro drugs profiles to patients. Next, find out whether the in-vitro profile predicts the in-vivo response/non-response to a given drug in a given patient. Finally, further substantiate that mitochondrial function parameters in lymphocytes reflect the long-term response to the drugs. The results we obtained by investigating these four issues justify a larger scale study.

## Methods

### Subjects

Healthy controls (n = 40) were recruited from the staff and faculty of the Beer-Sheva Mental Health Center, Faculty of Health Sciences, Ben-Gurion University of the Negev, Beer-Sheva and from Rambam Health Care Campus, Haifa, Israel. Hospitalized and outpatients, recruited from Beer-Sheva Mental Health Center, were evaluated according to Diagnostic and Statistical Manual of Mental Disorders V (DSM-V). Patients were diagnosed with either schizophrenia (SZ; n = 48) or bipolar-disorder (BD; n = 27) and severity of symptoms was assessed by the Clinical Global Impression (CGI) and the Brief Psychiatric Rating Scale (BPRS). All groups (patients and controls) were matched for age and gender as much as possible. The study was approved by institutional review board (IRB). All subjects gave informed consent for participation in the study. Inclusion criteria for the controls were: subjects aged 18–60 years who signed an informed consent form. Exclusion criteria: pregnant women and subjects with a history of neurodegenerative and metabolic diseases that require drug treatment, or any kind of malignancy and substance use. For control subjects—in addition to the above, no psychiatric disorders or family history of psychiatric disorders.

### Lymphocyte separation and treatment

Blood (40–50 ml) was withdrawn and lymphocytes separated on Ficoll-Paque gradient (Lymphoprep™, Axis-Shield POC AS, Oslo, Norway) by centrifugation (Supplementary Methods 1). Seven drugs were studied: typical antipsychotic drugs (haloperidol and perphenazine), new generation antipsychotic drugs (risperidone & olanzapine) and clozapine and the mood stabilizers [lithium and valproic acid (VPA)] (Sigma-Aldrich, St. Louis, MO, USA). Doses within the therapeutic range^28^ were tried and optimal concentrations were chosen (Table 1S). For protein and mRNA levels cells were centrifuged at 300xg and the pellet washed by centrifugation at 300×*g* with phosphate buffer saline (PBS) for RNA and with PBS containing 10 mM ethylenediaminetetraacetic acid (EDTA) for protein extraction. The dry pellet was frozen at − 80 °C until use.

**Table 1:** Demographic and clinical data.

	Controls	Patients		
		Untreated (SZ/BD)	Treated for one month (SZ/BD)	Chronically-treated (SZ/BD)
No. of subjects	40	20	11	55
Gender males/females	19/21	5/15	2/9	19/36
Origin Ashk.J/Non.Ask.J/Other	16/15/9	8/10/2	4/6/0	25/27/3
Age average ± SD (range)	33.4 ± 8.7 (19–54)	38.5 ± 15.4 (16–67)	40.7 ± 16.2 (16–67)	39.5 ± 12.5 (19–66)
Age of onset ± SD (range)		29.2 ± 9.4 (16–48)	32.1 ± 8.9 (20–46)	24.4 ± 9.0 (19–50)
Duration of illness* (range)		10.10 ± 8.7 (0–32)	9.6 ± 7.1 (0.5–21)	15.5 ± 10.7 (0.6–43)
No. of patients diagnosed SZ /BD		18/2	9/2	30/25
Haloperidol equivalents^a^ (mg/day)			12.8 ± 7.7	7.9 ± 7.3
			Responders (n = 7)/non-responder (n = 4)	
BPRS		47.2 ± 9.3	32.0 ± 11.2/45.3 ± 10.0*	46.4 ± 12.1
CGI-S		4.6 ± 0.6	2.7 ± 1.3/4.4 ± 0.5**	3.8 ± 1.5

*p < 0.0005; **p < 0.02.

^a^Treated for one month: haloperidol n = 2; Lithium n = 3; Olanzapine n = 2; Perphenazine n = 1; Risperidone n = 2; Valproic acid n = 1. Chronically treated: Clozapine n = 16; Haloperidol n = 6; Lithium n = 14; Olanzapine n = 4; Perphenazine n = 1; Risperidone n = 6; Valproic acid n = 8.

### Mitochondrial respiration

The effect of antipsychotics and mood stabilizers on mitochondrial respiration in human lymphocytes was carried out in the Seahorse XF-24 analyzer (Seahorse Biosciences, North Billerica, MA) (Supplementary Methods 1). Parameters obtained were: basal-, adenosine triphosphate (ATP)-linked-, maximal- and reserve capacity-oxygen consumption rates (OCR), proton leak, and non-mitochondrial respiration. Results were normalized to cell count/well.

### Protein levels

Total protein was extracted from lyzed lymphocytes and assessed as described previously^29^. Levels of 14 different proteins were measured by indirect Enzyme-Linked Immunosorbent Assay (ELISA) as previously described^30^ with minor modifications (Supplementary Methods 1). Assessed proteins were those associated with the respiratory chain [complex I—the subunits NADH:Ubiquinone Oxidoreductase Core Subunit V1 (NDUFV1), NADH:Ubiquinone Oxidoreductase Core Subunit V2 (NDUFV2), NADH:Ubiquinone Oxidoreductase Core Subunit S1 (NDUFS1); complex II—succinate dehydrogenase A (SDHA); complex IV—Cytochrome c oxidase subunit 2 (COX2); apoptosis—B-cell lymphoma 2 protein (Bcl-2), Bcl2 Associated X protein (BAX), Caspase 3 (CASP3); mitochondrial network dynamics—optic atrophy 1 (dynamin-like GTPase) (OPA1), Mitofusin 1 (MFN1), Dynamin related protein 1 (Drp1), Fission 1 (FIS1) and autophagy—Beclin1, SQSTM1-sequstosome 1 (p62)]. The antibodies source and dilutions are summarized in Table 2S.

### mRNA levels

mRNA levels were assessed following RNA extraction by quantitative reverse transcriptase—polymerase chain reaction (qRT-PCR) (Supplementary Methods 1 and 3S).

All methods were performed in accordance with the relevant guidelines and regulations.

### Statistical analysis

Results were analyzed for normal distribution using the Kolmogorov–Smirnov test. As data mostly distributed normally and in order to address the issue of multiple comparisons, differences between the effect of drugs were analyzed by one-way or two-way ANOVA followed by post-hoc Fisher's Least Significant Difference (LSD) test if main effect or interactions were significant (P < at least 0.05). Correlations were analyzed by the Pearson correlation test. The STATISTICA software version 12 (StatSoft, Tulsa, Oklahoma, USA) was used for all analyses. Results exceeding ± 2SDs were excluded.

### Ethics approval and consent to participate

The study was approved by institutional review board (IRB) of Soroka Medical Center Beer-Sheva No. 5223 and of Rambam Health Care Campus No. RMB 0124-17. All subjects gave informed consent for participation in the study.

## Results

### Subjects

Two groups of patients: (1) Chronically (in-vivo) treated (SZ and BD) for at least two years (n = 55) who received the psychotropic drugs that were investigated in the current study. (2) Untreated patients for at least one month (n = 20) 11 of whom could be recruited again after a month of drug treatment and a group of healthy controls (n = 40). Table 1 summarizes the demographic and clinical data of the subjects. Response/non-response in the one month treated patients was defined by CGI-I < 3 / ≥ 4, respectively, and by overall clinical impression. Significant differences were observed for the duration of illness (F_2,73_ = 4.273 p = 0.04) among the patient groups. CGI-S and BPRS were significantly different between responding and non-responding patients treated for one month (F_1,10_ = 6.696 p = 0.02) and (F_1,21_ = 0.697 p = 0.0005) respectively. No significant difference was observed for all other parameters in all groups.

We further analyzed all data using age, age of onset of disease and disease duration as continuous predictors in ANOVA and did not find significant influences. Sex, origin, family history of psychotic disease and smoking were used as co-factors in ANOVA and no significant interaction with mitochondrial parameters was observed in any group.

### In-vitro effect of psychotropic drugs on mitochondrial parameters in healthy subjects

**Figure 1:**
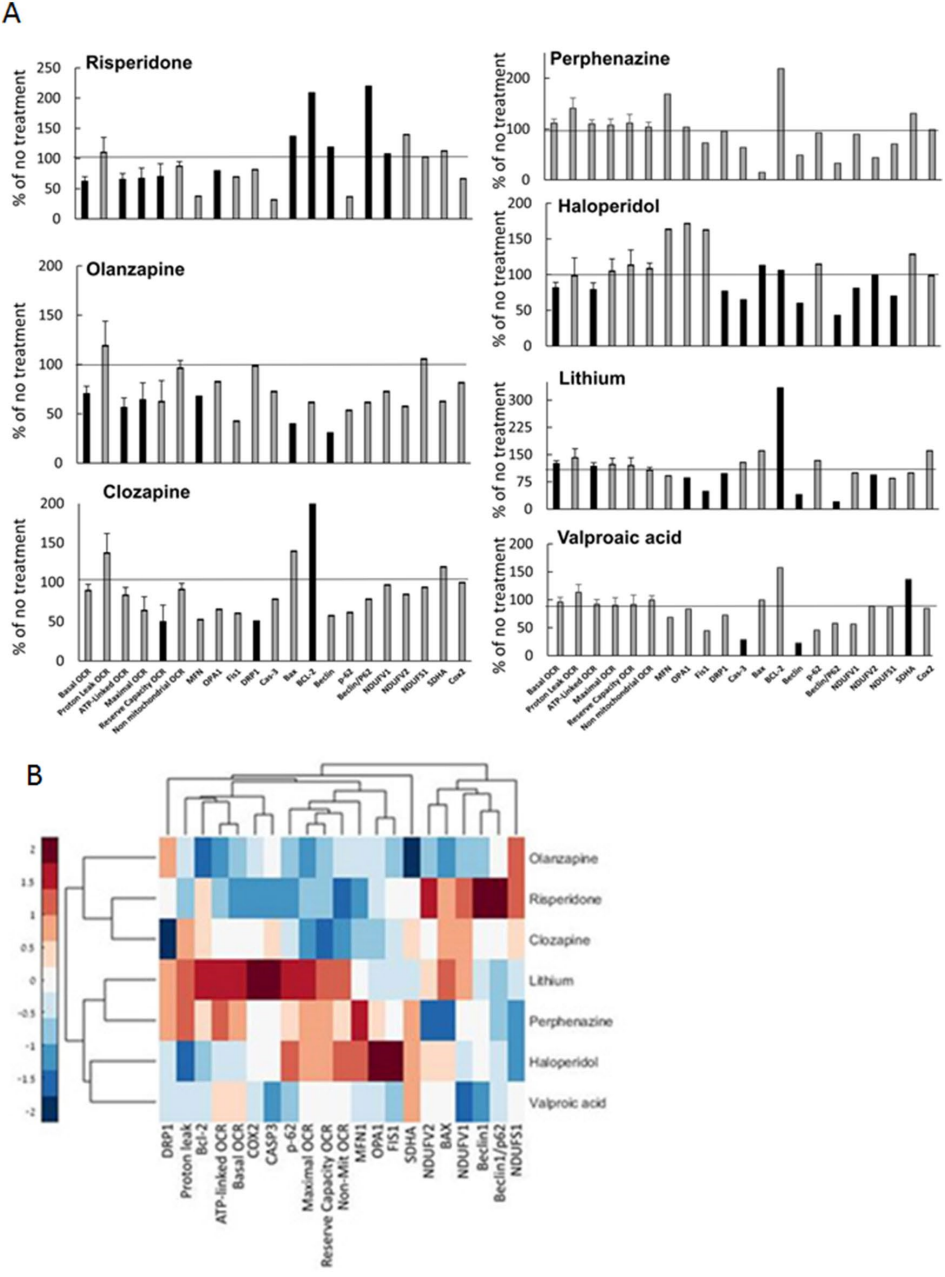
A specific mitochondrial fingerprint is observed for each of the psychotropic drugs studied in healthy controls. A. Isolated lymphocytes were treated for 24 h in-vitro with therapeutically relevant concentration of each drug. Values are means ± SEM expressed as percent of control (without drug). For each drug n = 12–21 subjects. The data were analyzed by one-way ANOVA followed by LSD post-hoc analysis, P < at least 0.05 if main effect was significant (P < at least 0.05) and are means ± SEM expressed as percent of change of the untreated corresponding subject. Black bars denote statistically significantly effects. B. Hierarchical clustering dendogram and heat map summarizing the effect of psychotropic drugs on mitochondrial functional parameters. The typical antipsychotic drugs perphenazine and haloperidol clustered separately from the atypical drugs olanzapine, risperidone and clozapine. The mood stabilizers lithium and VPA clustered with the typicals, albeit in different subgroups.

Six mitochondrial respiration parameters and levels of 14 proteins related to mitochondrial function were analyzed. The proteins included were those of the electron transport chain (ETC)—complex I subunits (NDUFV1, NDUFV2, NDUFS1), complex II subunit SDHA and complex IV subunit COX2; apoptosis—BAX, Bcl-2, CASP3, mitochondrial fission/fusion—OPA1, MFN1, Drp1, FIS1 and autophagy—Beclin1, p62. Most of the included proteins were reported abnormal in one or both disorders^31–38^. By comparing mitochondrial parameters with and without drug treatment, a specific profile of the effect of each of the drugs was obtained (Fig. 1A), demonstrating significant increase or decrease (P < at least 0.05 by LSD post–hoc in variables that their main effect in ANOVA was P < at least 0.05) or no change.

For mitochondrial respiration parameters we found that olanzapine, haloperidol and risperidone significantly decreased basal- and ATP-linked-OCR, while lithium significantly increased these parameters. Clozapine and risperidone significantly decreased mitochondrial reserve capacity OCR. Olanzapine and risperidone significantly decreased mitochondrial maximal respiration. VPA and perphenazine did not affect any mitochondrial respiration parameter.

Proteins significantly decreased by lithium were complex I subunit NDUFV2, three mitochondrial fission/fusion proteins OPA1, FIS1, Drp1, and the autophagy-associated protein Beclin1 as well as Beclin1/p62 ratio, while the anti-apoptotic protein Bcl-2 was increased. VPA significantly increased levels of the complex II protein SDHA, while decreasing levels of the pro-apoptotic protein CASP3 and Beclin1. Clozapine increased levels of Bcl-2, while decreasing levels of Drp1. Olanzapine decreased levels of CASP3, the fusion protein MFN1 and Beclin1. Risperidone significantly increased the levels of both pro- and anti-apoptotic proteins BAX and Bcl-2, respectively, as well as levels of Beclin1 and Beclin1/p62 ratio, while decreasing levels of OPA1. Haloperidol increased levels of Bcl-2 and BAX. However, this drug significantly decreased the levels of the ETC complex I subunits NDUVF1, NDUVF2 and NDUVS1, CASP3, Drp1, Beclin1 and Beclin1/p62 ratio.

Hierarchical clustering dendorgram revealed a specific fingerprint for each of the drugs with typical antipsychotics clustering separately from the atypical drugs. The mood stabilizers clustered with the typical drugs, albeit in different subgroups, with lithium clustering with perphenazine and VPA with haloperidol (Fig. 1B).

In contrast with the results obtained for protein levels, psychotropic drugs treatment failed to induce any significant change at the level of mRNA expression of the assessed proteins (Supplementary Material 1 and Table S4).

### In-vitro psychotropic drugs induce similar changes in mitochondrial parameters in healthy controls and untreated patients

**Figure 2:**
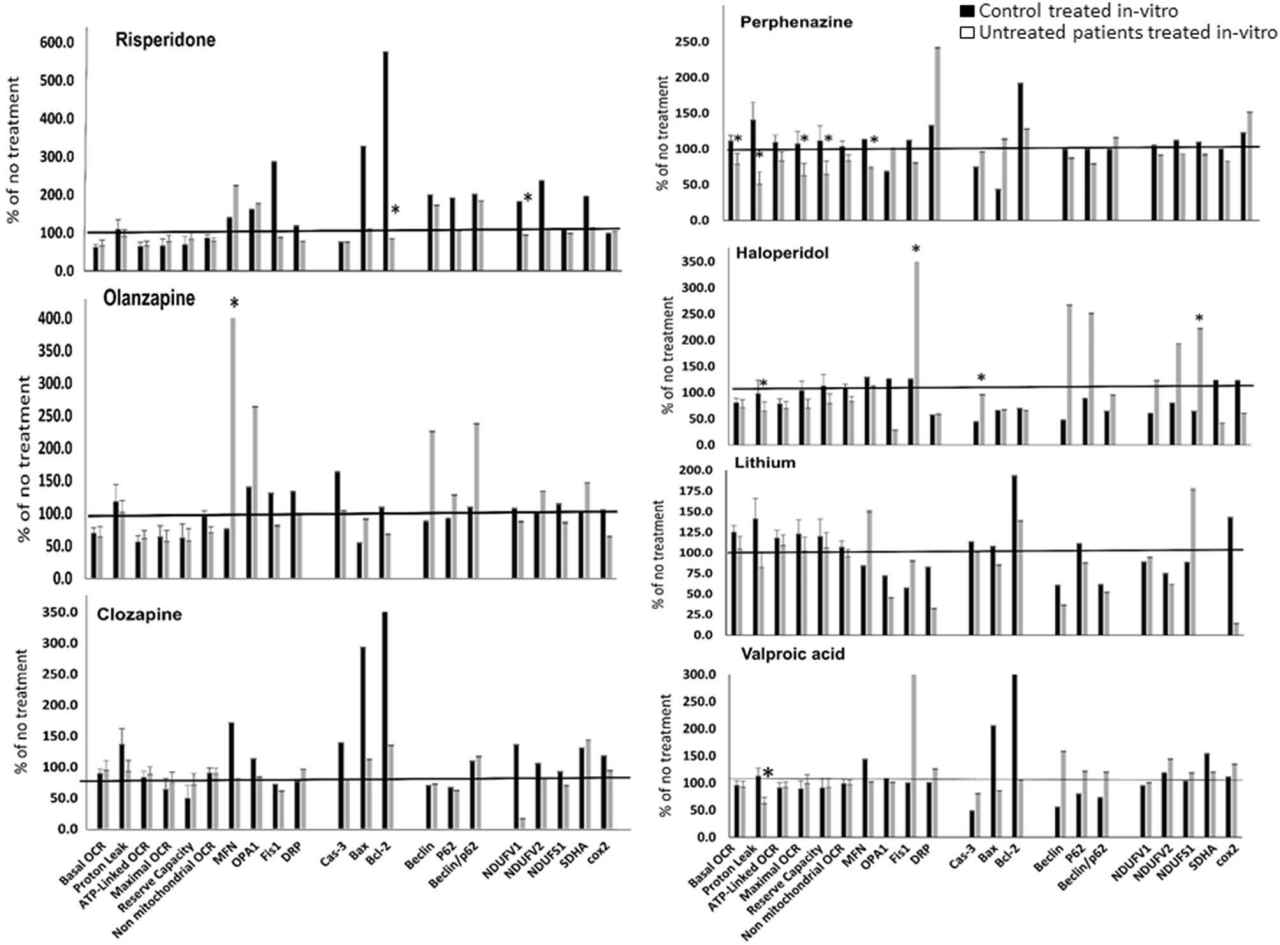
Different psychotropic drugs show a similar pattern of in-vitro effect on mitochondrial parameters in healthy subjects and untreated patients with psychosis. Some of the drugs showed significant differences between the groups for a few parameters. Isolated lymphocytes were treated for 24 h in-vitro with therapeutically relevant concentration of each drug. The data were analyzed by one-way ANOVA followed by LSD post-hoc analysis, *P < at least 0.05 if main effect was significant (P < at least 0.05) and are means ± SEM expressed as percent of change of the untreated corresponding subject. n = 8–21 subjects for each group and treatment.

Drug-induced changes were compared between healthy controls and untreated patients with psychosis for each drug and each parameter. There was no difference in the in-vitro effect of lithium, clozapine, olanzapine and risperidone on any of the respiration parameters between the two groups. The effect of VPA, haloperidol and perphenazine on proton-leak was significantly different between untreated patients and controls, reducing this process in patients. Perphenazine also differently affected basal- maximal- and reserve capacity-OCR in untreated patients and controls (Fig. 2). As for the mitochondria-related protein levels, no difference was observed in the in-vitro effect of VPA and clozapine between the two diagnostic groups. Lithium and olanzapine also showed a similar profile in both groups except for one protein in each, COX2 and MFN1, respectively. Risperidone's profile was different between the groups in two proteins, NDUFV1 and Bcl-2 and that of perphenazine in MFN1. The most pronounced difference between the two groups was observed following haloperidol exposure, albeit only for five out of the 14 proteins assessed (NDUFS1, SDHA, CASP3, OPA1 and FIS1). These findings suggest that by and large, the mechanism of action of the drugs on mitochondrial parameters in-vitro is similar in both groups (P < at least 0.05 by LSD post-hoc in variables that their main effect in ANOVA was P < at least 0.05).

### Significant correlations between a drug-induced in-vitro and in-vivo effects in a given patient before and after short-term treatment

**Figure 3:**
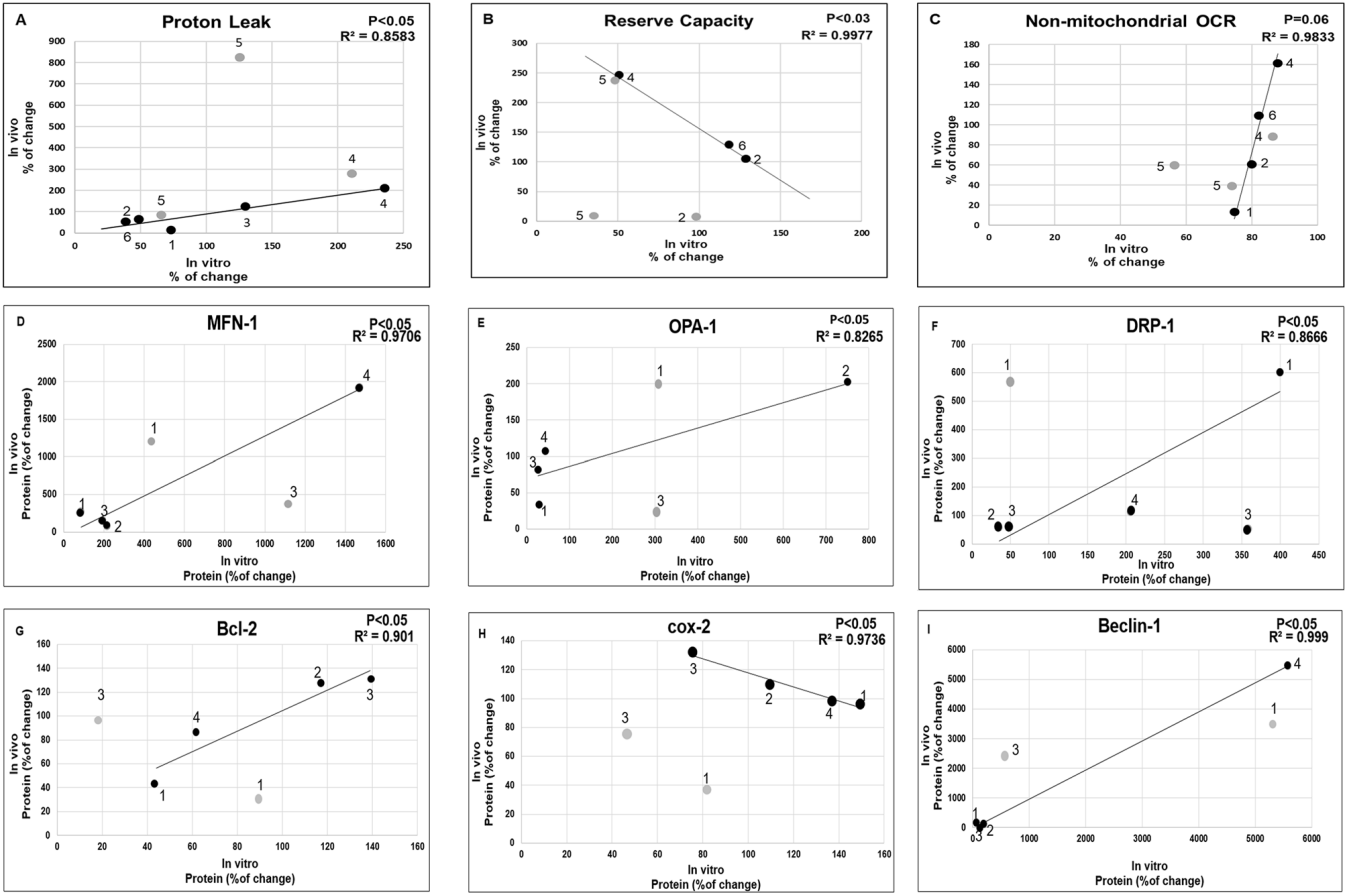
Significant correlations, analyzed by Pearson's correlation analysis, were obtained between in-vitro drug-induced effects in lymphocytes isolated from untreated patients and the in-vivo short-term treatment-induced effects with a given drug in a given patient. Namely, the x axis presents data of in-vitro effect of drugs prior to commencement of treatment (% of change from no drug) and the y axis presents data of in-vivo effect of the same drug after about a month of treatment (% of change from before treatment) in a given patient. Black dots—responders; grey dots—non-responders. Numbers indicate the drug: (1) perphenazine, (2) lithium, (3) olanzapine, (4) risperidone, (5) haloperidol and (6) VPA.

Lymphocytes from an untreated patient with psychosis were first assessed for the in-vitro effect of a given drug and again about a month following treatment (in-vivo). Each patient was assigned to receive one of the study drugs chosen by the psychiatrist to be used for the patient's treatment. Clozapine was not included since it is not routinely prescribed as a first choice drug. Unfortunately, the amount of blood withdrawn from some of the patients was insufficient to analyze all parameters. Therefore, the effect of all six drugs was monitored for mitochondrial respiration parameters and of haloperidol, lithium, risperidone and olanzapine for protein levels. For each of the mitochondria-related parameters a correlation between the in-vitro and the in-vivo drug-induced changes was analyzed for responding and non-responding patients. In responding patients, a significant correlation between the in-vitro and the in-vivo drug-induced effects was observed for nine out of 20 parameters, including proton-leak, reserve capacity- and non-mitochondrial-OCR, COX2, Bcl-2, MFN1, OPA1, Drp1 and Beclin1. In contrast, in most patients who failed to improve clinically following treatment (non-responders) no correlation was observed for any of the parameters (Fig. 3), suggesting relevance of the in-vitro effects of the drugs to their in-vivo effects.

Although preliminary, it is of interest to note a qualitative similarity (increase, decrease or no change) between risperidone's profile in controls, and the drug's in-vitro effect in two untreated, eventually responding patients on 60% (12/20) of the parameters studied . In contrast, in an eventually non-responder there was only 25% similarity. Another patient who eventually did not respond to haloperidol exhibited only 20% similarity, while an eventually lithium responder showed 54% similarity to the drugs' profile in controls.

### Long- but not short-term antipsychotic treatment normalizes mitochondria-related parameters in patients

**Figure 4:**
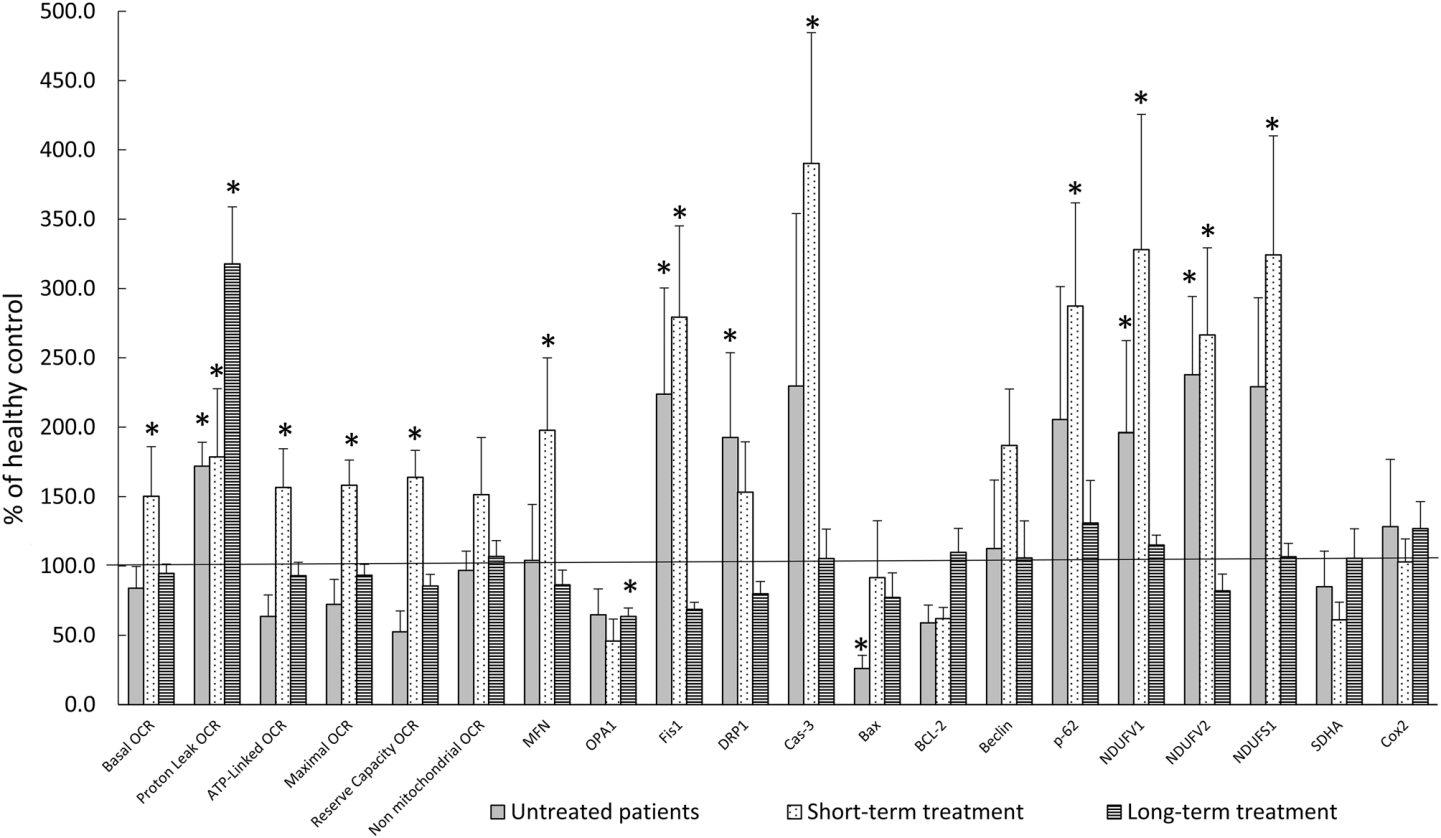
Long- but not short-term in-vivo treatment by psychotropic drugs normalized mitochondrial parameter in patients with psychosis responding to treatment. VPA was excluded from the protein levels analysis since only one sample was available. Data were analyzed by one-way ANOVA followed by LSD post hoc analysis and are means ± SEM. *P < at least 0.05 vs. healthy controls.

In untreated, eventually responding patients, basal levels of 13 out of the 20 mitochondrial parameters assessed, differed from those of healthy subjects. Basal-, ATP-linked-, maximal- and reserve capacity-OCR were significantly lower, while proton leak and levels of the proteins NDUFV1, NDUFV2 NDUFS1, CASP3, MFN1, FIS1, Drp1 and p62 were significantly higher in these patients as compared to controls (Fig. 4). Short-term treatment (about one month) caused a significant increase above control levels in all but one of the mitochondrial respiration parameters, in NDUFV1, NDUFV2, NDUFS1, CASP3, FIS1, and p62, while a decrease in OPA1 and SDHA levels (Fig. 4). On the contrary, long-term treatment (several years) revealed a similar profile of mitochondrial respiration parameters in patients and healthy controls except for proton leak values, which were higher. Notably, although the comparison was carried out in a pool of patients treated with a variety of drugs (as patient number on a given drug in the short-term treatment was too small for individual drug analysis), the generalized result obtained indicates a duration-dependent mode of action of the drugs. Perphenazine was excluded from the comparison since only one patient in each group was treated with this drug. We did not find correlation between the mitochondrial parameters and dose of medication in the chronically treated patients. Due to the small sample size of the short term treated group and the variety of drugs they received the analysis of such a correlation is statistically unjustified.

## Discussion

In quest for a rational basis to predict the appropriate drug for an individual, recent intensive research focuses on developing tools predicting individual’s response to psychotropic drugs based on genetic fingerprints (pharmacogenomics)^39–42^. Studies of the genetic basis of the response to treatment in SZ suggest that DNA sequences related to drug metabolism and target sites can predict treatment response (reviewed in Ref.^43^). The authors advocated that pharmacogenetics of this disorder may assist personalized treatment and lead to a molecular-based sorting of response to adverse effects of psychotropic drugs. They further alleged that "*clinicians may soon be using a patient's genotype to decide initial choice of antipsychotic drug treatment in SZ*". Ten years later these expectations have only partially ripened. Namely, quantification and interpretation of drug concentrations in blood to optimize pharmacotherapy, designated therapeutic drug monitoring (TDM), has been extensively developed^44^. However, TDM does not indicate the optimal drug for a given patient. An alternative emerging approach for personalized medicine suggests mining existing clinical trials' data to build statistical models to predict drug response^45^. One such example is a study in which machine learning was applied to patient-reportable pre-treatment data from 334 patients of the European First Episode Schizophrenia Trial in order to provide useful insight into individualized outcome trajectories and to optimize treatment selection^46^. Such an approach does not take into account individual biological factors, while the genetic approach ignores factors affecting drug response such as environmental parameter, epigenetic status and microbiome. Indeed, it was recently claimed that "*Environmental factors (age, sex, diet, alcohol use, hormonal status, general health) and co-medication are usually more important factors than inherited determinants of drug metabolism and response*"^47^. Together, genetic and environmental factors culminate in functional manifestations, which were the focus of the present study. In particular, based on multiple lines of evidence suggesting that mitochondrial dysfunction is a key component in the neurobiology of neuropsychiatric disorders including SZ and BD^7,16,48^ and a target of psychotropic drugs^22,35,49^, we studied twenty mitochondrial function parameters. We addressed two main issues: the **relevance** of mitochondrial function parameters to psychotropic treatment and the **predictability** of these parameters for personalized medicine in psychosis.

Here we show an in-vitro drug-specific mitochondria-related effect profile in healthy controls-derived lymphocytes. This, along with disparate clustering of typical *vs.* atypical antipsychotic drugs support the relevance of mitochondrial function parameters as a tool for personalized treatment prediction. The profiles are comprised of respiration parameters and mitochondrial function-related proteins but not of their mRNA, which was not affected by the 24 h drug treatment. The latter is in line with a previous study reporting lack of haloperidol effect on mRNA expression of complex I subunits in a human neuronal cell line^50^ and with the comprehension that mRNA and protein levels are not always affected to the same extent by intra and/or extra-cellular processes^51,52^. Alternatively, this lack of effect could indicate that short-term in-vitro exposure to psychotropic drugs affects post-transcriptional mechanisms.

The in-vitro profile of drugs' effect obtained in untreated patients was similar to that in healthy controls. By-and-large our data demonstrate high similarity in the extent of change following drug exposure between the two diagnostic groups. Seemingly, this similarity is counterintuitive given the strong evidence of mitochondrial deregulation in psychotic disorders. This apparent conundrum may be conciliated if the protein-domain targeted by a drug is not impaired in patients. Supporting the latter are the findings that antipsychotic drugs affect complex I-driven respiration to the same extent in lymphocyte-derived cell lines of SZ patients and healthy controls although complex I activity is abnormal in the patients^35^. As for the typical drugs, about 25% of the parameters were affected only in the patients, with haloperidol affecting mitochondrial-related proteins, while perphenazine—respiration parameters. Drug-specific induced differences between patients and controls may result from intrinsic disease-dependent differences in drugs' interaction sites. Haloperidol and perphenazine are prone to participate in redox reactions but in a different manner. Haloperidol has oxidation potency^53^ while perphenazine is an oxidation substrate^54^. Our data suggest that the patients are more susceptible to oxidoreductive load. Nonetheless, the resemblance in drug effect profiles between untreated patients and controls supports the notion that mitochondrial drug effect profiles may be useful in predicting response to psychotropic drugs.

The predictability is further supported by the significant correlations between the in-vitro (before treatment) and in-vivo (after ~ 1 month treatment) drug response in treatment-responding patients as opposed to lack of correlation in non-responding patients. The correlation was observed for all five drugs used to treat these patients and for almost half of the studied parameters, implying that the in-vitro response of several parameters can foresee the in-vivo effect and indicate a predictive relationship. The lack of significant correlation for the rest of the parameters may reflect either their irrelevance to the predictive profile, or be due to the small sample size of patients we managed to recruit. A greater sample size is essential in order to support the potential of the in-vitro drug profile as a predictor for in-vivo drug-response. Although anecdotic, our results showing that individuals' *in*-*vitro* response profile to two drugs in eventually responding untreated patients resembles the profile of controls, as opposed to eventually non-responders, are in favor of the predictability potential of mitochondrial function parameters.

The findings of the in-vivo drug treatment effects in patients provide additional evidence for mitochondria being a relevant target for psychotropic drugs. Notably, in responding patients, short-term treatment was not sufficient to ameliorate mitochondrial dysfunction, while a prolonged drug treatment restored mitochondrial function. This is in line with numerous reports of duration-dependent mode of action of drugs^55–57^. Long-term treatment did not normalize the high proton leak in patients indicating an increased uncoupling between oxidative phosphorylation and ATP synthesis, and corroborates with dissipation of the mitochondrial membrane potential (Δѱ_m_) in SZ^58,59^. Protons leak through uncoupling proteins (UCPs) on the mitochondrial inner membrane. Interestingly, association of mitochondrial UCP genes with SZ was reported^60,61^.

## Conclusions

In conclusion, the present proof-of-concept study was aimed to set the ground for using lymphocyte mitochondrial function parameters as a tool to predict the drug of choice for a given patient with psychosis (personalized medicine). Using an array of mitochondrial parameters we found an in-vitro drug–specific effect-profile and suggest its applicability by demonstrating a correlation between pre-treatment in-vitro and in-vivo effect of antipsychotic drugs on mitochondrial parameters only in patients eventually responding to treatment. An additional novel finding is that antipsychotic treatment amends the levels of mitochondrial parameters to almost those of healthy controls, but prolonged medication is required.

Our study is not devoid of limitations including the small sample size in the untreated patients' group and the retrospectivity of the chronically treated patients study. The current results pave the road and calls for a big data replication study in order to embed this tool into real-world healthcare. Regardless, this study contributes to the understanding of the role of mitochondria in psychosis and its treatment.

## Supplementary information

Supplementary file 1

## Data Availability

The datasets used and/or analyzed during the current study are available from the corresponding author on reasonable request.
