# Supplementary material for: Mitochondrial function parameters as a tool for tailored drug treatment of an individual with psychosis: a proof of concept study
Source: Sci Rep. 2020 Jul 23;10:12258. doi: 10.1038/s41598-020-69207-4 (PMC7378204; doi:10.1038/s41598-020-69207-4)
Supplement: Supplementary file 1 [file 41598_2020_69207_MOESM1_ESM.docx]

Supplementary Materials

Mitochondrial function parameters as a tool for tailored drug treatment of an individual with psychosis: a proof of concept study

Tamara Bar-Yosef, Wessal Hussein, Ofer Itzhaki, Odeya Damri, Limor Givon, Carmit Marom, Vlada Gurman, Joseph Levine, Yuly Bersudsky, Galila Agam, Dorit Ben-Shachar

Supplementary Methods:

Lymphocyte separation and culturing

Lymphocytes were separated on Ficoll-Paque gradient (Lymphoprep™, Axis-Shield POC AS, Oslo, Norway) by centrifugation at 400xg for 30 minutes at 4°C. Lymphocytes, 0.75x10^6^ or 1.5x10^6^ cells/well, were plated in 96- or six-well plates for mitochondrial respiration measurements and protein levels, respectively, and grown in RPMI growth medium (Sigma-Aldrich Ltd, St. Louis, MO, USA) containing 10% human serum, 2 mM L-glutamine, 100 U/ml streptomycin/penicillin (Biological Industries, Beit Haemek, Israel) at 37Cº, with 5% CO_2_. Cells obtained from healthy subjects and untreated patients were incubated with or without psychotropic drugs in duplicates for 24 hours. Seven drugs were studied: typical antipsychotic drugs (haloperidol and perphenazine), new generation antipsychotic drugs (risperidone & olanzapine) and clozapine and the mood stabilizers [lithium and valproic acid (VPA)] (Sigma-Aldrich, St. Louis, MO, USA). Doses within the therapeutic range^31^ were tried and optimal concentrations were chosen (Table 2). For protein and mRNA levels cells were harvested, centrifuged at 300xg and the pellet washed by centrifugation at 300xg with PBS for RNA and with PBS containing 10 mM EDTA for protein extraction. The dry pellet was frozen at -80°C until use.

## Mitochondrial respiration

Mitochondrial was carried out in the Seahorse XF-24 analyzer (Seahorse Biosciences, North Billerica, MA). Due to the non-adhesive character of lymphocytes Seahorse XF Analyzer plates were coated with poly-D-lysine 50 µg/ml (Sigma-Aldrich, St. Louis, MO, USA). Oxygen consumption rate (OCR) was measured every 10 min for 2 h according to the manufacturer's instructions. Optimized concentrations of respiration inhibitors were: 2.5 µM oligomycin, 0.8 μM carbonyl cyanide 4-trifluoromethoxy phenylhydrazone (FCCP) and 1 μM rotenone. Data from the Seahorse XF Apparatus were collected using the XF Reader software from Seahorse Biosciences (North Billerica, MA).

Indirect Enzyme-Linked Immunosorbent Assay (ELISA)

Protein samples [(100 µg/100 µl of 10 mM phosphate buffered saline (PBS)] were added to flat-bottom Maxisorp 96-well plates (NUNC, Thermo-Fisher Scientific, Waltham, MA, USA) incubated for 2 h at 37^0^C, dried, blocked with 1% bovine serum albumin (BSA) in PBS (blocking buffer) for 1 h at 37^0^C, washed and then incubated with primary antibody (1 h, 37^o^C), washed 3 times and incubated with secondary antibody (1 h, 37^0^C). Antibody reactivity was assessed by incubation with 3,3’,5,5’-tetramethylbenzidine (TMB) (Sigma-Aldrich, St. Louis, MO, USA) for 30 min at room temperature. The reaction was stopped with 11.6 M HCl and absorbance measured at 450 nm.

RNA extraction

RNA was extracted using Tri Reagent (Sigma-Aldrich, St. Louis, MO, USA), followed by purification using phenol:chloroform:isoamyl alcohol and isopropranol. Samples were kept at -20°C overnight, followed by high speed centrifugation and washing of the RNA pellet with ethanol. The pellet was then dissolved in DEPC-treated water and analyzed for quantity and quality spectrophotometrically at 230 nm, 260 nm and 280 nm. RNA integrity depicted in the form of three bands corresponding to 28S, 18S and 5S RNA was assessed by electrophoresis in a 1% agarose/formaldehyde gel stained with ethidium bromide.

qRT-PCR

Five 5µg RNA was reverse transcribed with random primers using cDNA synthesis kit (Versotm ABgene, Thermo scientific, Waltham, MA). The resulting single strand cDNA was amplified by real-time PCR (STRATAGENE, ThermoFisher Scientific) using gene-specific designed primers (Suppl. Table 1) and SYBR green dye (SensiMix SYBR kit, London, UK). Real-time PCR program consisted of three steps: first – 15 min at 95^o^C for denaturation and enzyme activation, followed by 4^o^C amplification cycles of 15 seconds at 95^o^C, 30 seconds at 60^o^C and 5 seconds at 72^o^C, for denaturation, annealing and extension, respectively, and lastly, a final extension step at 72°C for 5 min. Template control (NTC) reactions in which cDNA is substituted with DNase/RNase-free water was included in each real-time PCR run to screen for possible contamination. For primers' sequence see Table S3.

Table 1S: Final Drugs' concentrations

| **Drug** | **Concentration** |
| --- | --- |
| **Perphenazine** | 2.4 ng/ml |
| **Olanzapine** | 80 ng/ml |
| **Haloperidol** | 10 ng/ml |
| **Risperidone** | 60 ng/ml |
| **Clozapine** | 60 ng/ml |
| **Valproic acid** | 80 ng/ml |
| **Lithium** | 42 ng/ml |

Table 2S: Proteins and antibodies used for ELISA

| **2º Antibody dilution** | **2º Antibody** | **1º Antibody dilution** | **1º Antibody** | **Protein** |
| --- | --- | --- | --- | --- |
| 1:15000 | Anti-rabbit IgG  Abcam | 1:2500 | Rabbit polyclonal Sigma-Aldrich | 24kDa (NDUFV2) |
| 1:10000 | Anti-rabbit IgG  Abcam | 1:1000 | Rabbit polyclonal Sigma-Aldrich | 51kDa (NDUFV1) |
| 1:10000 | Anti-rabbit IgG  Abcam | 1:1000 | Rabbit polyclonal Sigma-Aldrich | 75kDa (NDUFS1) |
| 1:10000 | Anti-mouse IgG  Abcam | 1:2000 | Mouse monoclonal  Santa Cruz Biotechnology (Santa Cruz, CA, USA) | SDHA  (Succinate Dehydrogenase) |
| 1:1000 | Anti-mouse IgG  Abcam | 1:500 | Mouse monoclonal Abcam | Cox2  (Cytochrome c oxidase subunit 2) |
| 1:1000 | Anti-mouse IgG  Abcam | 1:500 | Mouse monoclonal Abcam | Bax |
| 1:2000 | Anti-rabbit IgG  Abcam | 1:500 | Rabbit polyclonal  Cell Signaling | Caspase-3 |
| 1:500 | Anti- rabbit IgG  Abcam | 1:500 | rabbit polyclonal  Santa Cruz Biotechnology (Santa Cruz, CA, USA) | Bcl2 |
| 1:10000 | Anti-rabbit IgG  Abcam | 1:1000 | Rabbit polyclonal  Cell Signaling | Beclin |
| 1:5000 | Anti-rabbit IgG  Abcam | 1:1500 | Rabbit polyclonal  Abcam | p-62 (Sequestosome-1) |
| 1:5000 | Anti-mouse IgG  Abcam | 1:250 | Mouse monoclonal  Santa Cruz Biotechnology (Santa Cruz, CA, USA) | OPA-1 (Optic atrophy 1) |
| 1:5000 | Anti-rabbit IgG  Abcam | 1:500 | rabbit polyclonal  Santa Cruz Biotechnology (Santa Cruz, CA, USA) | MFN1  (Mitofusin-1) |
| 1:5000 | Anti-mouse IgG  Abcam | 1:250 | Mouse monoclonal  Santa Cruz Biotechnology (Santa Cruz, CA, USA) | DRP1  (Dynamin-related protein 1) |

Table 3S: List of primers

| **Gene** | **GenBank** | **Primer sequence** |
| --- | --- | --- |
| β –actin- FW | NC_000007.14 | ACCAGTTCGCCATGGATGA |
| β –actin - REV | NC_000007.14 | AAGCCGGCCTTGCACAT |
| NDUFV1-FW | NC_000011.10 | GTAATCCCAGCACTTTGG |
| NDUFV1-REV | NC_000011.10 | TAGAGACAGGGTTTCACC |
| NDUFV2-FW | NC_000018.10 | CATTCAGGTCTGCACTAC |
| NDUFV2-REV | NC_000018.10 | GTTTGTCAGGTGTAGTCTC |
| NDUFS1-FW | NC_000002.12 | GTGAGATTGCAGGAGTAG |
| NDUFS1-REV | NC_000002.12 | GATATTCCCAGACAGTTCAG |
| COX1-FW | NC_001224.1 | CTTATCCTACCAGGCTTC |
| COX1-REV | NC_001224.1 | CATAGCTCAGACCATACC |
| SDHA-FW | NC_000005.10 | GGTTTATGGAGCGATACG |
| SDHA-REV | NC_000005.10 | CTCTTCCTTCTCGGATCT |
| OPA1-FW | NC_000003.12 | GATAGTTCTCGGGAGTTTG |
| OPA1-REV | NC_000003.12 | GGTACAGCCTTCTTTCAC |
| MFN1-FW | NC_000003.12 | CGAACAGCACACTATCAG |
| MFN1-REV | NC_000003.12 | TTGGTCTTCCCTCTCTTC |
| DRP1-FW | NC_000012.12 | GTACAGTGATCGGAAAGG |
| DRP1-REV | NC_000012.12 | CTTCAGCCTCTGTTATC C |
| FIS1-FW | NC_000007.14 | GAAGAAAGATGGACTCGTG |
| FIS1-REV | NC_000007.14 | CGTCTCCTTCAGGATTTG |
| CASP3-FW | NC_000004.12 | CGITGTAGAAGTCTAACTGG |
| CASP3-REV | NC_000004.12 | CATCAACACCACTGTCTG |
| BAX-FW | NC_000019.10 | TGGACATTGGACTTCCTC |
| BAX-REV | NC_000019.10 | AAAGATGGTCACGGTCTG |
| BCL2-FW | NC_000018.10 | CCTTTGTGGAACTGTACG |
| BCL2-REV | NC_000018.10 | GAGCAGAGTCTTCAGAGA |
| LC3B-FW | NC_000016.10 | GAGAAGACCTTCAAGCAG |
| LC3B-REV | NC_000016.10 | GCTTCTCACCCTTGTATC |
| BECN1-FW | NC_000017.11 | CAGGAGAGGAGCCATTTA |
| BECN1-REV | NC_000017.11 | GCACTTTCTGTGGACATC |
| p62-FW | NC_000005.10 | CATCCATCAGTACCCTTG |
| p62-REV | NC_000005.10 | GTAGAGGTTCCACTTCATC |

Table 4S: Effect of drugs on mRNA levels in healthy controls
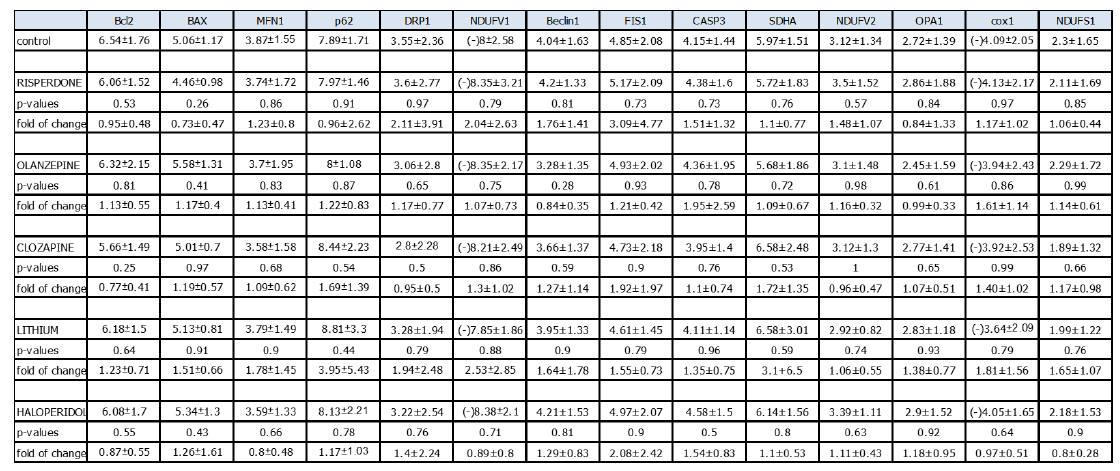


Data are expressed as means ± SD of ΔΔCT and of fold of change, n=11 subjects.
